# Ulcerative Colitis-Induced Colorectal Carcinoma: A Deleterious Concatenation

**DOI:** 10.7759/cureus.22636

**Published:** 2022-02-26

**Authors:** Ana P Rivera, Gabriela Vanessa Flores Monar, Hamza Islam, Sri Madhurima Puttagunta, Rabia Islam, Sumana Kundu, Surajkumar B Jha, Ibrahim Sange

**Affiliations:** 1 Research, Universidad Americana (UAM) Facultad de Medicina, Managua, NIC; 2 Research, Universidad Central del Ecuador, Quito, ECU; 3 Research, Faisalabad Medical University, Faisalabad, PAK; 4 Research, Dr. P.S.I. (Pinnamaneni Siddhartha Institute) Medical College, Chinoutpalli, IND; 5 Research, R G Kar Medical College, Kolkata, IND; 6 Research, Jinan University School of Medicine, Guangzhou, CHN; 7 Research, K. J. Somaiya Medical College, Hospital and Research Center, Mumbai, IND

**Keywords:** inflammatory bowel disease, colorectal carcinoma, uc, colonoscopy, colon cancer screening, colon cancer surveillance, colon cancer, ibd, ibd associated cancer, ulcerative colitis

## Abstract

Inflammatory bowel disease (IBD) is a chronic inflammatory gastrointestinal ailment that encompasses Crohn's disease (CD) and ulcerative colitis (UC). UC is an idiopathic, chronic inflammatory condition of the colonic mucosa that begins in the rectum and progresses proximally in a continuous way over a portion of the entire colon. Chronic inflammation is linked to cancer, and IBD-related chronic colonic inflammation raises the risk of colorectal cancer. Chronic inflammation has been linked to cancer, and chronic colonic inflammation caused by IBD increases the risk of colorectal cancer (CRC). When CRC arises in people with IBD, unlike sporadic CRC, the lesions are difficult to identify due to mucosal alterations produced by inflammation. The total prevalence of IBD-associated CRC is increasing due to the rapidly increasing frequency of IBD. Screening and surveillance colonoscopy in IBD patients is considered to allow for the early diagnosis of dysplasia and cancer, improving the prognosis of IBD-related CRC by giving patients proactive therapy. This article has reviewed literature pertaining to the mechanisms related to CRC development in UC and its clinical and therapeutic implications.

## Introduction and background

Ulcerative colitis (UC) is an idiopathic, chronic inflammatory disorder of the colonic mucosa, which starts in the rectum and generally extends proximally in a continuous manner through part of or the entire colon [1]. UC was first described in the 1800s by Samuel Wilks and is considered one of the two forms of idiopathic IBD alongside CD [2]. Both UC and CD are more frequent in the developed world, especially in North America and Western Europe, although their prevalence is increasing in Asia [3]. In the Western population, the incidence and prevalence of IBD have increased in the past 50 years, up to 8-14/100,000 and 120-200/100,000 people, respectively, for UC [4]. Despite a rise in the frequency of UC in various age groups in recent decades, most UC patients are between the ages of 30 and 40 when the diagnosis is made [4]. UC has equal incidence in males and females and is more prevalent in newly industrialized countries. It has been associated with an urban lifestyle, pollution, diet, antibiotics, better hygiene, and fewer infections [5]. Alterations in the colonic microbiome and disruption of the intestinal mucosa have been pointed out to be possible risk factors for the development of UC [6]. An autoimmune response underlines the pathogenesis of UC carried out by T-helper 2 (TH2) cells, which disrupt the colon's superficial lining, leading to the development of ulcers and crypt abscesses [7]. The most prominent clinical features of the disease include abdominal pain, diarrhea, and hematochezia. However, more than one-third of patients with IBD are affected by extraintestinal manifestations or extraintestinal complications beyond the intestinal expression of the disease. The most common signs include arthropathies, mucocutaneous, ophthalmological manifestations, and conditions affecting the hepatobiliary system [8]. UC is diagnosed based on clinical presentation, endoscopic assessment (gold standard), and histologic criteria [9]. With these studies, it is possible to identify the involvement of the rectum as the primary site of inflammation and the distinctly confluent form of inflammation that ends with an abrupt demarcation and transition into normal colonic mucosa [5]. The first-line treatment for UC management is 5-aminosalicylic acid [10]. Corticosteroids may be added if first-line therapy fails to control the disease, and infliximab can be added to induce and sustain remission [10]. Patients with severe or nonresponsive UC should be hospitalized, and intravenous corticosteroids should be given. Surgical intervention is indicated for severe disease [10]. UC is characterized by chronic inflammation, which may lead to the accumulation of high levels of pro-inflammatory cytokines within the colonic mucosa and thus to dysplastic lesions and cancer. Patients with UC have a 2.4-fold higher risk of CRC than the general population [11]. The purpose of this article is to underline the pathogenic relationship between UC and CRC, explore the clinical association between UC and CRC, emphasize the existing screening guidelines for the prevention of CRC in UC patients.

## Review

**Figure 1:**
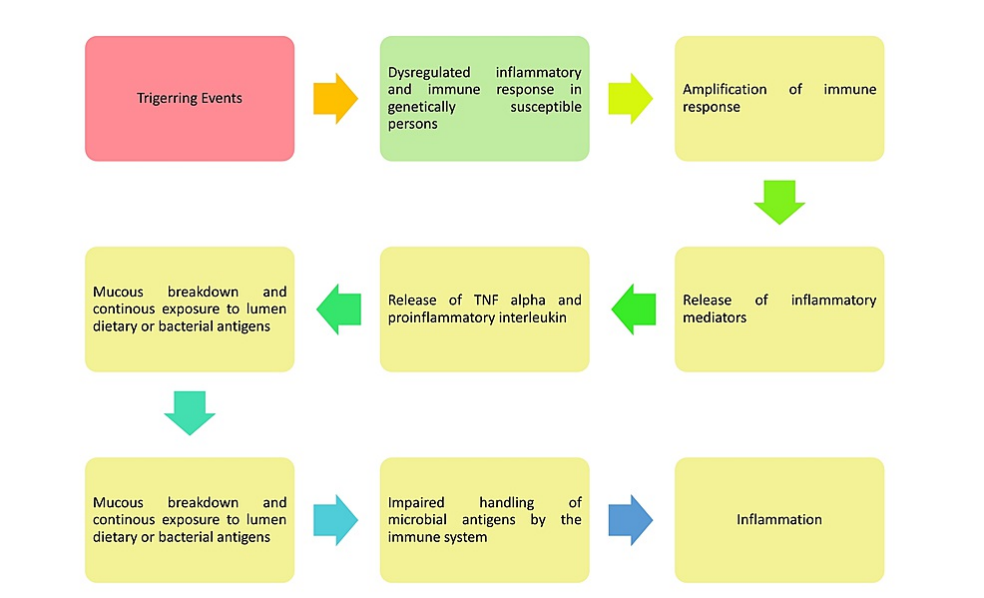
Pathophysiology of inflammatory events in IBD IBD: inflammatory bowel disease

UC is a chronic illness of the large intestine that is becoming more common worldwide. This disease affects nearly one million people in the United States and Europe, with much more worldwide [1]. UC is a chronic immune-mediated inflammatory disorder of the large intestine that is commonly linked with inflammation of the rectum but also spreads proximally to include other parts of the colon [1]. Symptoms of an inflamed rectum, such as bleeding, urgency, and tenesmus, define the first presentation of UC [2]. The illness can appear at any moment; however, there is a preferred age distribution of onset that peaks between 15 and 30 years [1]. The most common pattern of disease activity is relapsing and remitting, with active illness symptoms alternated by periods of remission. Some people with UC have chronic disease activity despite the diagnosis and medical therapy, and a minority proportion of patients develop fulminant illness, a rapid-onset progressive form of colitis [3]. With UC, the immune system misidentifies the colon's lining as a foreign entity and targets it, which eventually causes intestinal damage. As the body attempts to repair the damage, the inflammation and continual cell replacement might result in a mutation that leads to cancer. Figure 1 illustrates the pathogenesis pathway of events leading to inflammation in IBD. 

Shared pathways and mechanisms

The pathophysiology of UC is a generally recognized concept that proposes a complex contribution of environmental and host variables that enhance the vulnerability to developing UC [4]. Disease onset is initiated by events that disrupt the mucosal barrier, disrupt the healthy balance of the gut microbiota, and inappropriately trigger gut immune responses. The adenylate cyclase 7 (ADCY7) gene has the highest genetic relationship with UC. ADCY7 is one of 10 enzymes that convert ATP to the ubiquitous second messenger cAMP [5]. Furthermore, numerous UC-specific genes are involved in controlling epithelial barrier function. The significant growth in UC prevalence in newly industrialized nations shows that environmental variables have a role. A new urban lifestyle accompanies westernization, exposure to pollution, dietary change, availability of antibiotics, improved cleanliness, and fewer illnesses, all of which are seen as general contributory factors [6]. In altering the healthy gut microbiota, one of the significant impacts of dysbiosis in UC is believed to be a loss in epithelial health or a state of epithelial malfunction, which further primes innate vulnerability to UC. The epithelium is either changed or impaired secretion or physical abnormalities. Histologic evidence of sub-epithelial inflammation in the presence of epithelial disruption suggests that a compromised epithelial barrier is a pathogenic component for UC [6]. Dysbiosis causes a decrease in short-chain fatty acid synthesis, which is necessary for epithelial energy provision, mucus formation, and colon growth [1].

**Figure 2:**
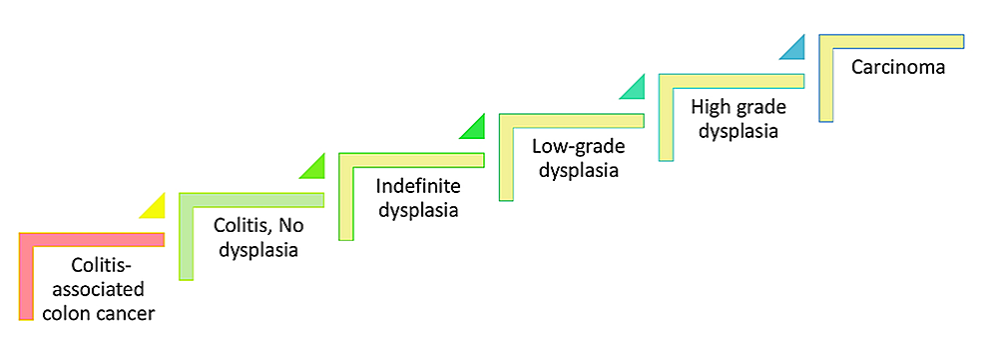
Etiology of colitis-associated CRC CRC: colorectal cancer

Previous research has revealed that people with UC have an elevated risk of CRC; thus, screening recommendations for this patient group are in place. In individuals who have not been diagnosed with UC, a physician can use a clinical history to distinguish between the various etiologies of persistent diarrhea [8]. The existence of constitutional symptoms and extraintestinal signs, notably arthritis and skin lesions, in a patient with established UC may indicate the disease's severity [9]. In individuals with gastrointestinal symptoms, finger clubbing raises the chance of UC, whereas the lack of clubbing does not. Colonoscopy, proctosigmoidoscopy, and biopsy are the most often used diagnostics to diagnose UC. Loss of the usual vascular pattern, friability, exudates, ulcerations, and granularity are characteristic alterations [10]. Due to the inflammation-induced alterations in the colonic mucosa, malignancy is difficult to detect by endoscopy in UC patients; tumor borders are indistinct, and tumors tend to grow in various shapes [11]. Figure 2 depicts the pathophysiology of colitis-associated CRC progressing to carcinoma.

Literature review and epidemiological implications of screening for CRC in UC

Several studies examining the prevalence of CRC in IBD patients have discovered an increased risk of developing this malignant tumor compared to the control group study subjects. Indeed, CRC accounts for roughly 10% to 15% of all fatalities in IBD patients. However, the accurate degree of their elevated risk for the disease is still being debated due to differences between earlier and current epidemiological data. According to a Swedish population-based investigation, the incidence of CRC was 5.7 times higher (95% CI: 4.6-7.0) than the average rate in one of the first cohort studies of 3117 individuals with UC (diagnosed from 1922 to 1983) from the Uppsala region in Sweden. A Canadian cohort study of 5529 individuals with IBD tracked between 1984 and 1997 and included in the Manitoba Health database revealed a comparable rise in CRC prevalence in both UC and CD. In rectal cancer, UC patients had a higher incidence ratio than the general population, whereas CD patients did not [11]. Recent population-based research shows that the level of risk is substantially less and limited to certain geographic areas. An analysis of 692 individuals from Olmsted County, Minnesota, USA, between 1940 and 2001 found that the standardized incidence ratio for CRC was not significantly greater in patients with UC and CD than in the non-affected population. The only statistically significant increase in risk was a two-fold rise in instances of prolonged UC. Other population-based studies in Hungary and Denmark found either no increased risk or a marginally more significant risk of CRC in persons with UC and CD [12].

There is comparative evidence regarding the actual risk of CRC in CD patients. However, in the Asia-Pacific area, more long-term data on the cumulative risk attributed to UC and CD are necessary [12]. In the Asia-Pacific region, the prevalence of CRC in UC patients ranges from 0.3% to 1.8%, and a recent nationwide study conducted by the Korean Association for the Study of Intestinal Diseases (KASID) discovered a cumulative incidence of UC-associated CRC comparable to that of western countries. The results exhibit a high degree of variability, in part, to regional differences in CRC incidence among nations. The yearly risk of CRC for patients with IBD has been estimated to be four to five instances per 1000 person-years in the United States and the United Kingdom. However, the risk appears to be significantly lower in Scandinavia and other countries; two cases per 1000 person-years. The cause of this regional disparity is unknown. Still, various variables, including genetic factors, nutrition, usage of chemoprevention, better surgical therapies, enhanced colonoscopy monitoring, or a combination of both, can be implicated [12]. Numerous studies use different methodologies for estimating risk, which is likely to influence the outcomes. Several studies, for example, provide the cumulative risk of developing CRC in a specific community of IBD patients without stratifying the patients based on various risk factors. In contrast, others report a standardized incidence ratio without providing information on the lifetime risk [11-12].

Many studies have been conducted to find a correlation between UC and CRC. Patients with UC at high risk of colorectal neoplasia (CRN) were provided screening by magnifying chromocolonoscopy in this prospective trial from Shivakumar BM et al.; physicians examined the prevalence of neoplastic lesions. The study contained 29 (70.7%) of 41 eligible individuals with a median age of 46 recruited in the surveillance program, and 41 colonoscopies were performed for 42 months [13]. The average illness duration was 10 years. Sixteen people (55.1%) had severe colitis. On the first screening, five (17.2%) had low-grade dysplasia (LGD), and three had high-grade dysplasia (HGD) (10.3%). One of these three accepted proctocolectomy right away, one underwent adenocarcinoma surgery and one declined surgery [13]. Three additional LGD were discovered during 12 follow-up colonoscopies in nine individuals. It was determined that in individuals with long-standing UC, HGD and subsequent adenocarcinoma might be diagnosed with meticulous follow-up; however, acceptance of monitoring and later therapy is unsatisfactory. There is evidence that screening and monitoring systems can help identify neoplasia in UC as seen in Table 1.

**Table 1:** Population data table with studies implicating epidemiology of colitis-associated CRC UC: ulcerative colitis; CRC: colorectal carcinoma; CRN: colorectal neoplasia; IBD: inflammatory bowel disease; HGD: high-grade dysplasia; CC: colon cancer

References	Design	No. of Cases	Study Parameters	Conclusion
Shivakumar BM et al. [13]	Cohort	41	Screening CRN among UC patients in India	With diligent monitoring, HGD and eventual cancer can be diagnosed. Screening and monitoring strategies can help discover neoplasia in UC patients.
Kim BJ et al. [14]	Cohort	7061	Incidence of CRC in patients with UC in Korea	In Korea, the cumulative incidence of UC-associated CRCs was found to be equivalent to that of Western countries. Because the total prevalence of UC-associated CRC in Korea may be increasing, comprehensive surveillance colonoscopy and constructive chemoprevention should be encouraged to allow for early identification and treatment.
Rutter MD et al. [15]	Cohort	68	Determining colonoscopy markers for cancer risk could allow patient risk stratification	In UC, macroscopic colonoscopy characteristics can help predict the likelihood of neoplasia. Characteristics of prior or continuing inflammation indicate an elevated risk.
Jess T et al. [16]	Cohort	47,374	CRC risk in a nationwide cohort of 47,374 Danish patients with IBD over a 30-year period	A UC or CD diagnosis no longer appears to enhance patients' risk of CRC, while subsets of UC patients remain at greater risk. The lower risk of CRC from 1979 to 2008 might be attributed to better therapy for IBD patients.
Korelitz BI et al. [17]	Cohort	115	Whether histological inflammation persists despite endoscopic mucosal healing serves to increase the risk of CC or HGD	Progression to HGD or CC in individuals with long-standing severe UC was more common in those with chronic histological inflammation in the absence of gross mucosal illness. Our findings support the inclusion of histological inflammation in the concept of mucosal healing, as well as this endpoint as a suitable therapeutic aim due to the danger of increased dysplasia and CC.

In the study by Kim BJ et al., the KASID examined 7061 cases of UC between 1970 and 2005 and discovered 26 instances of CRC. In individuals with UC, the overall frequency of CRC was 0.37%. Furthermore, the estimated cumulative risk of UC-associated CRCs was 0.7% for patients with UC for 10 years, 7.9% for patients with UC for 20 years, and 33.2% for patients with UC for 30 years [14]. The average age at the time of CRC diagnosis was 49.6 years, and the average duration of UC before CRC development was 11.5 years. Many UC-associated CRCs were detected after they had progressed to an advanced stage; however, the scene at diagnosis was lower in individuals who adhered to medical therapy [14]. In Korea, the cumulative incidence of UC-associated CRCs was equivalent to that of Western countries. Because the overall prevalence of UC-associated CRC in Korea may be increasing, rigorous surveillance colonoscopy and constructive chemoprevention should be encouraged to allow for the early diagnosis and treatment of UC-associated CRCs in Korea.

Following up on an earlier study that found a link between inflammation severity and neoplasia risk, a case-control study by Rutter MD et al. was conducted to test for colonoscopy indicators of CRN risk in UC. The physician matched each patient diagnosed with neoplasia between 1988 and 2002 with two non-dysplastic colitis controls. Researchers gathered data on post-inflammatory polyps, scarring, strictures, backwash ileitis, a shorter, tubular, or featureless colon, severe inflammation, and normal-looking surveillance colonoscopies. The number of cases (68) and controls (136) were matched [15]. On univariate analysis, cases were significantly more likely to have post-inflammatory polyps (odds ratio (OR) 2.14 (95% CI 1.24-3.70)), strictures (OR 4.22; 1.08-15.54), shortened colons (OR 10.0; 1.17-85.6), tubular colons (OR 2.03; 1.00-4.08), or segments of severe inflammation (OR 3.38; 1.41-10.13). A macroscopically normal-looking colonoscopy (OR 0.38; 0.19-0.73), post-inflammatory polyps (2.29; 1.28-4.11), and strictures (4.62; 1.03-20.8) remained significant after multivariate analysis [15]. After a normal-looking colonoscopy, the risk of CRC was no different from that of matched general population controls after five years. Analysts concluded that in UC, macroscopic colonoscopy characteristics could help predict the likelihood of neoplasia. Previous or ongoing inflammatory characteristics indicate an elevated risk and, in this group, monitoring frequency should be reduced to five years.

Jess T et al. studied CRC risk in a nationwide cohort of 47,374 Danish patients with IBD over 30 years. CRC formed in 268 people with UC and 70 patients with CD during 178 million person-years of follow-up [16]. The overall risk of CRC among UC patients was equivalent to the general population (Relative Risk (RR), 1.07; 95% CI, 0.95-1.21). Patients with UC diagnosed in infancy or adolescence, those with a long duration of illness, and those with associated primary sclerosing cholangitis (PSC) were at higher risk [16]. The overall RR for CRC in UC patients fell from 1.34 (95% CI, 1.13-1.58) in 1979-1988 to 0.57 (95% CI, 0.41-0.80) in 1999-2008. The overall RR for CRC among CD patients was 0.85 (95% CI, 0.67-1.07), and it did not alter over time. A UC or CD diagnosis no longer appears to enhance patients' risk of CRC while subsets of UC patients remain at greater risk. The lower risk of CRC from 1979 to 2008 might be attributed to better therapy for IBD patients.

A study by Korelitz BI et al. was done to determine if histological inflammation that persists beyond endoscopic mucosal healing increases the risk of colon cancer (CC) or HGD in patients with severe UC. The inclusion criteria were satisfied by 68 of the 115 individuals with long-standing UC reviewed. Group 1 consisted of 20 patients, whereas Group 2 consisted of 48 individuals [17]. Researchers counted the number of times each patient's endoscopic appearance was expected, but biopsies revealed inflammation. In all, 31.2% of the colonoscopies done on the complete cohort of 68 individuals had histological disease activity in the absence of endoscopic illness. When the colonoscopy revealed no gross disease activity, histological disease activity was more prevalent in Group 1 patients than in Group 2, 88% versus 59%. Only 3/20 (15%) of patients in Group 1 had a colonoscopy with no evidence of disease activity (no endoscopic or histological activity), compared to 37/48 (77%) of patients in Group 2. Only 3.3% of colonoscopies in Group 1 had no histological inflammation, compared to 23% in Group 2 [17]. Progression to HGD or CC in individuals with long-standing severe UC was more common in those with chronic histological inflammation in the absence of gross mucosal inflammation. These findings support the inclusion of histological inflammation in the concept of mucosal healing and another endpoint as a suitable therapeutic aim due to the danger of increased dysplasia and CC.

Risk factors

Women and men are almost equally affected by UC. Among the risk factors are: CRC risk factors in patients with inflammatory bowel disease increase with disease duration, the severity of colitis, a family history of CRC, coexisting PSC, UC pancolitis, UC left-sided colitis, CD colitis, active inflammation, and grade of inflammation. See Table 2.

**Table 2:** Risk factors for UC UC: ulcerative colitis

Non – modifiable risk factors	
Age	UC generally develops before the age of 30, however, it can occur at any age.
Race or ethnicity	Caucasians are at the highest risk of contracting UC. People of Ashkenazi Jewish origin are at an even higher risk of developing UC.
Genetics	People with a family history of UC are at a greater risk of developing the disease (parent, sibling, or child with UC).
Modifiable risk factors	
Environmental factors	Reacting to things in the environment such as bacteria or chemicals can cause uncontrollable inflammation in the gastrointestinal system.
Diet and lifestyle	While they are less prevalent risk factors for UC, greater consumption of polyunsaturated fatty acids may lead to digestive health difficulties. A sedentary lifestyle and smoking are also risk factors for general health that affect your gut health.

In a study by Lakatos L et al., the goal was to determine risk factors for and the epidemiology of CRC in UC patients in Veszprem province. They analyzed all UC patients' pertinent epidemiological and clinical data in Veszprem province using the 30-year IBD database [18]. Data from 723 UC patients (m/f: 380/343) were analyzed. In these UC patients, the rate of familial illness was 5.2%, and the prevalence of non-CRC-associated colectomies was 3.7%. CRC was detected in 13 individuals (m/f: 6/7, 13/8564 person-years). The average age of onset of UC in the 13 patients with UC-CRC was 34.5 (13-61) years, which was 4.1 years younger than the age of onset in UC patients without CRC. The mean age of UC-CRC patients at CRC diagnosis was 50.9 (27-70) years (duration of UC: 16.5 +/- 8.2 years), over 15 years younger than the norm in Hungary's sporadic CRC group. Eight patients survived (survival: 67.9 (10-163) months), four patients died from CRC (survival: 8.0 months), and one died from an unrelated reason after being diagnosed with CRC for 10 years [18]. Longer illness duration, chronic continuous disease, more extensive colitis, iron deficiency or chronic anemia, PSC, and dysplasia in the biopsy were all risk factors for CRC. Longer illness duration, severe colitis, PSC, and dysplasia were associated with an elevated risk in a logistic regression model. After 10 years of illness, the cumulative chance of getting CRC was 0.6% (95% CI: 0.2-1.0%), 5.4% (95% CI: 3.7-7.1%), and 12.6% after 32 years (95% CI: 7.0-18.2%). CRC detected via a surveillance colonoscopy was related to a higher chance of survival (p = 0.04) [18]. Most UC patients had an increased cumulative risk of CRC, although it was lower than in Western European and North American investigations. When compared to random CRC patients, CRC developed around 15 years sooner. Long illness duration, severe colitis, iron shortage or chronic anemia, dysplasia, and PSC appear to be key risk factors for CRC in UC patients.

Jess T et al. conducted a case-control study of risk factors and protective factors for colorectal dysplasia and cancer in patients with IBD in two well-described IBD cohorts from Copenhagen County, Denmark, and Olmsted County, Minnesota [19]. The study comprised 43 instances of neoplasia that were matched based on six criteria. Conditional logistic regression was used to calculate the probability of neoplasia for each variable. PSC (odds ratio (OR) 6.9, 95% CI: 1.2-40), percentage of disease course with clinically active disease (OR 1.2, 95% CI 0.996-1.4), and > or = 1 yr. of continuous symptoms (OR 3.2, 95% CI 1.2-8.6) was associated with neoplasia, whereas a borderline association with a median number of small-bowel X-rays (OR 1.3, 95% CI 0.96-1.6) was observed [19]. Observations did not reveal protective effect of frequency of physician visits (OR 1.4, 95% CI 0.96-2.0), number of colonoscopies (OR 1.4, 95% CI 1.0-2.1), cumulative dose of sulfasalazine (OR per 1,000 g, 1.1, 95% CI 1.0-1.3) and mesalamine (OR per 1,000 g, 1.3, 95% CI 0.9-1.9), or partial intestinal resections (OR 1.5, 95% CI 0.3-7.1). CRN was more common in IBD patients with PSC, severe long-standing illness, and X-ray exposure. The protective impact of careful monitoring, colonoscopy, and 5-aminosalicylate therapy appeared doubtful.

In a study by Gupta RB et al., the aim was to determine whether the intensity of microscopic inflammation over time is an independent risk factor for neoplastic development in UC. Of the 418 individuals who satisfied the inclusion criteria, 15 had advanced neoplasia (HGD or CRC), and 65 developed any neoplasia (LGD, HGD, or CRC) [20]. Multivariable analysis revealed substantial associations between histologic inflammation over time and progression to advanced neoplasia (hazard ratio (HR), 3.0; 95% CI: 1.4-6.3 for IS-mean; HR, 3.4; 95% CI: 1.1-10.4 for IS-bin; and HR, 2.2; 95% CI: 1.2-4.2 for IS-max). It was concluded that in individuals with long-standing UC, the degree of microscopic inflammation over time is an independent risk factor for developing advanced CRN.

A study piloted by Nieminen contained patients who have had IBD for a long time and are at a higher risk of developing CRC. Previous research suggests that the intensity of inflammation is a separate risk factor for CRC in UC. To better target dysplasia surveillance in IBD, scientists evaluated the relevance of histological inflammation as a risk factor for colorectal dysplasia or CRC. Between 1996 and 2008, researchers identified 183 IBD patients with dysplasia or CRC by integrating our hospital patient registry and pathology database [21]. The control group was drawn from our IBD patient registry. Histologically severe inflammation was found in 41.4% of dysplasia patients and 24.1% of CRC patients, but only 4.3% of controls. In comparison to patients with no inflammation, those with severe inflammation had an OR of 31.8 (95%, CI: 15.6-64.9) for dysplasia or cancer. The OR was 2.6 among individuals with mild to severe inflammation (95% CI: 1.6-4.1). Disease duration raised the yearly chance of dysplasia or CRC by 4.5%. Coexisting PSC did not increase the risk. In contrast, usage of thiopurines (OR = 0.09, 95 percent CI: 0.02-0.33) and 5-aminosalicylic acid (OR 0.17, 95% CI: 0.017-1.01) did [21]. The degree of inflammation and the length of the condition both raise the risk of dysplasia and CRC. PSC was not recognized as a risk factor. Researchers observed that using thiopurines significantly reduces the risk of CRC (refer to Table 3). These findings can be used to improve dysplasia surveillance in IBD patients.

**Table 3:** Studies highlighting risk factors of colonic inflammation leading to CRC UC: ulcerative colitis; CRC: colorectal cancer; IBD: inflammatory bowel disease; CRN: colorectal neoplasia; PSC: primary sclerosing cholangitis

References	Design	No. of Cases	Study Parameters	Conclusion
Lakatos L et al. [18]	Cohort	723	Calculated the incidence and standardized incidence and mortality rate ratios of CRC among adult individuals with intact colons using Kaiser Permanente of Northern California's database of members with IBD and general membership data for the period of 1998 to June 2010 and evaluated trends in medication use and rates of cancer detection over time.	Our UC patients had a high cumulative risk of CRC, although it was lower than that reported in Western European and North American research. When compared to random CRC patients, CRC developed around fifteen years sooner. Long illness duration, severe colitis, iron shortage or chronic anemia, dysplasia, and PSC appear to be key risk factors for CRC in UC patients.
Jess T et al. [19]	Cohort	43	Conducted a nested case-control study of such factors in two well-described IBD cohorts from Copenhagen County, Denmark, and Olmsted County, Minnesota.	CRN was more common in IBD patients with PSC, severe long-standing illness, and x-ray exposure. The preventive impact of careful monitoring, colonoscopy, and 5-amino salicylate therapy appeared uncertain.
Gupta RB et al. [20]	Cohort	418	Determine whether the severity of microscopic inflammation over time is an independent risk factor for neoplastic progression in UC.	In individuals with long-standing UC, the degree of microscopic inflammation over time is an independent risk factor for developing advanced CRN.
Nieminen U et al. [21]	Cohort	183	Investigated the role of histological inflammation as a risk factor for colorectal dysplasia or CRC to better target dysplasia surveillance in IBD.	In conclusion, the degree of inflammation and the length of the condition both raise the risk of dysplasia and CRC. The presence of PSC was not recognized as a risk factor. We found that using thiopurines significantly reduces the risk of CRC. These findings can be used to improve target dysplasia monitoring in IBD patients.
Rutter M et al. [22]	Cohort	68	To determine if the severity of colonic inflammation is an important determinant of the risk of CRN.	The intensity of colonic inflammation is a key predictor of the risk of CRN in patients with long-standing severe UC. Endoscopic and histological grading of inflammation may allow for more accurate risk classification in monitoring systems.

Rutter M et al. conducted a study to investigate multiple possible risk factors for neoplasia; a case-control study of individuals with long-standing severe UC was designed. Between January 1, 1988, and January 1, 2002, all instances of CRN found by the monitoring program were investigated (n = 68). Each patient was paired with two controls from the same surveillance group (n = 136) [22]. Genders, colitis extent, age at onset, duration of colitis, and year of index surveillance colonoscopy were all matched. A simple score (0, standard; 1, quiescent/chronic inflammation; and 2, 3, and 4, mild, moderate, and severe active inflammation, respectively) was used to record segmental colonoscopy and histological inflammation. Other information gathered included a history of PSC, a family history of CRC, smoking, and drug use (mesalamine 5-aminosalicylic acid, azathioprine, and folate) [22]. Analysis of variance revealed a highly significant relationship between colonoscopy (OR, 2.5; P = 0.001) and histological inflammation scores and the probability of CRN. Other variables were not statistically significant. The histological inflammation score remained significant after multivariate analysis (OR, 4.7; P 0.001). The severity of colonic inflammation is a crucial predictor of the risk of CRN in patients with long-standing severe UC. Endoscopic and histological inflammation grading may allow for more accurate risk classification in monitoring systems [23].

Screening protocol

CRC can be prevented and detected early with a regular screening at 45. According to the US Preventive Services Task Force (USPTF), physicians should examine adults for CRC from 45 to 75. The Task Force suggests that individuals aged 76 to 85 should be evaluated by a medical professional. Stool tests, flexible sigmoidoscopy, colonoscopy, and CT colonography are among the CRC screening procedures recommended by USPTF [24]. See Tables 4-5.

**Table 4:** USPTF CRC screening protocol CRC: colorectal cancer; USPTF: US Preventive Services Task Force

Population	Recommendation	Grade
Adults aged 50-75 years	Screen all adults aged 50 to 75 years for CRC.	A
Adults aged 45 to 49 years	Screen adults aged 45 to 49 years for CRC.	B
Adults aged 76 to 85 years	Selectively screen adults aged 76 to 85 years for CRC, considering the patient’s overall health, prior screening history, and patient’s preferences.	C

**Table 5:** Recommended screening protocol as per USPTF HSgFOBT: high-sensitivity guaiac fecal occult blood test; FIT: fecal immunochemical test; CRC: colorectal cancer; USPTF: US Preventive Services Task Force

Recommended screening strategies as per USPTF
HSgFOBT or fecal immunochemical test (FIT) every year
Stool DNA-FIT every 1 to 3 years
Computed tomography colonography every 5 years
Flexible sigmoidoscopy every 5 years
Flexible sigmoidoscopy every 10 years + annual FIT
Colonoscopy screening every 10 years
Selectively screen adults aged 76 to 85 years for CRC
Discuss together with patients the decision to screen, taking into consideration the patient’s overall health status (life expectancy, comorbid conditions), prior screening history, and preferences.

Outcomes due to therapeutic compliance

The goal of the study by van Staa TP et al. was to assess the risk of CRC in individuals receiving aminosalicylates (5-ASA) for IBD. The study comprised 18,969 participants, 100 of whom developed CRC after exposure to 5-ASA [25]. Most of these cases had a history of UC (76 patients). In the case-control study, frequent users were shown to have a lower incidence of CRC compared to irregular users (crude OR 0.7 (0.44-1.03); adjusted OR 0.60 (0.38-0.96)). Regular sulfasalazine users with six to 12 prior prescriptions had an adjusted OR of 0.95 (0.22-4.11), those with 13-30 prior prescriptions had an adjusted OR of 0.41 (0.14-1.20), and those with more than 30 prior prescriptions had an adjusted OR of 0.77 (0.37-1.60). These values were 1.13 (0.49-2.59), 0.30 (0.11-0.83), and 0.31 (0.11-0.84) for mesalamine users, respectively [25]. These findings indicate that frequent 5-ASA administration is related to a slight decrease in the probability of CRC developing in UC.

A study by O’Connor et al. was performed to see the effect of mesalamine on the risk of CRN. Mesalamine was linked with a slight decrease OR of CRN (OR = 0.6, 95% CI, 0.4-0.9, P = 0.04). This impact was only shown in hospital-based trials, and it was only observed in the decrease of all CRN patients [26]. Patients given dosages more than 1.2 g per day had a decreased risk of CRN (OR = 0.5, 95% CI, 0.3-0.9, P = 0.02) than those given smaller doses [26]. This impact was also seen solely in hospital-based research. In contrast, independent of the trial setting, there was no reduction in the incidence of CRN in patients taking sulfasalazine (OR = 0.8, 95% confidence range, 0.5-1.2, P = 0.3). Mesalamine, particularly at dosages more than 1.2 g per day, reduces CRN incidence in IBD patient groups from referral centers. Sulfasalazine does not appear to lower the risk.

The therapy of UC entails the immediate treatment of all inflammatory symptoms, followed by the maintenance of remission. The treatment method is generally defined by the severity of the signs and the extent of intestinal involvement. Mesalamine (also known as 5-amino salicylic acid 5-ASA) is a first-line medicinal therapy that operates topically from the intestinal lumen to decrease the synthesis of several pro-inflammatory mediators [5]. Oral steroid treatment should be explored for individuals who do not improve with the maximum dosage of 5-ASA compounds or cannot handle the adverse effects. Prednisone is administered to these individuals in doses ranging from 40 to 60 mg per day. Full-dose treatment is continued until symptoms are entirely controlled (typically 10 to 14 days), at which point the dosage is gradually decreased by 5 mg per week [5]. When steroids promote remission, greater doses of 5-ASA are frequently necessary.

Limitations

CRC is a complex disease with multiple risk factors, including age, race or ethnicity, genetics, environment, food, and lifestyle. This article focuses solely on UC as a risk factor for CRC.

## Conclusions

UC is a chronic IBD in which the immune system's aberrant responses create inflammation and ulcers on the inner lining of the large intestine. The purpose of medical therapy is to cure inflammation and enhance a patient's quality of life by reducing diarrhea, bleeding, and discomfort. Long-term immunosuppressants or anti-inflammatory medicines are routinely utilized. Corticosteroids are the most often used treatment at first; however, they should only be used for a short period due to the adverse effects. The reason for investigating CRC mortality rather than just incidence is that frequent endoscopic screening may uncover very early types of CRC that would otherwise go undiagnosed. Chronic inflammation is the most important initiating factor in the development of CRC. Patients with severe colitis, PSC, CRC susceptibility, or childhood-onset UC have increased risk factors for CRC. Aside from early diagnosis of precancerous lesions or colonoscopy surveillance, another vital element is the increased usage of maintenance therapy with aminosalicylates and ursodeoxycholic acid in patients with PSC, indicating that mesalamine is likely a chemopreventive medication. As of today, based on existing research and international recommendations, the use of surveillance endoscopy and chemopreventive maintenance treatment in patients with UC should be advocated and reviewed. Some preventive strategies and therapy mentioned throughout the article need further research to confirm their effectiveness.
